# Editorial: Syntheses under extreme conditions

**DOI:** 10.3389/fchem.2024.1428895

**Published:** 2024-05-31

**Authors:** Jörn Bruns, Maxim Bykov, Gunter Heymann, Günther Thiele

**Affiliations:** ^1^ Institute of Inorganic Chemistry, University of Cologne, Cologne, Germany; ^2^ Institute of Inorganic and Analytical Chemistry, Goethe-University Frankfurt am Main, Frankfurt, Germany; ^3^ Department of General, Inorganic and Theoretical Chemistry, Universität Innsbruck, Innsbruck, Austria; ^4^ Fachbereich Biologie, Chemie und Pharmazie, Freie Universität Berlin, Berlin, Germany; ^5^ Institut für Anorganische und Analytische Chemie, Albert-Ludwigs-Universität Freiburg, Freiburg, Germany

**Keywords:** chemistry, extreme synthesis, high-pressure, diamond anvil cell, inorganic synthesis

“Extreme conditions” in chemistry greatly depend on the perspective. High temperatures, as commonly used in solid state chemistry may appear “extreme” to many chemists in other research areas, while a successful synthesis at room temperature of products previously only obtained at very high temperatures, might also be considered “extreme”. This Research Topic is dedicated to synthesis conditions that are beyond of what is typically feasible with standard laboratory techniques. It emphasizes the fundamental necessity of developing novel methods, training students in these techniques, and a curiosity-driven exploration of “How far can we go?” These approaches open novel pathways to previously unknown compounds and/or modifications, ultimately expanding our profound understanding of matter.

Undoubtedly, high-pressure syntheses are considered “extreme” by most chemists, and were employed in the articles of this Research Topic. To synthesize Dy_36_O_11_F_50_ [AsO_3_]_12_·H_2_O Schleid et al. followed an approach using suitable binary precursors in water at high-pressure conditions in gold ampoules. The structure comprises seven- and eightfold heteroleptic coordinated Dy^3+^ species, alongside [AsO_3_]^3−^ anions and crystal water trapped in large cavities.

Peña-Alvarez et al. report on the formation of sodium trihydride NaH_3_ from the reaction of NaH at high hydrogen pressures of about 30, 40, 50, and 75 GPa in a diamond anvil cell. The findings are supported by Raman experiments and are compared to first principle calculations.

NdRe_2_ was obtained by Hussein, Chuvashova et al. in the cubic MgCu_2_ structure type from reactions at high-pressure high-temperature conditions in a diamond anvil cell. The results illustrate the formation of Laves phase structures at such special conditions, and the authors discuss connections to nuclear waste materials.

Via a high-pressure high-temperature reaction within a diamond anvil cell, the novel lanthanum hydroxyborate La_2_B_2_O_5_(OH)_2_ was obtained. The structure comprises discrete [BO_3_]-units and three crystallographically independent lanthanum positions: one in ninefold, one in tenfold and one in twelvefold coordination, respectively. Besides the synthesis and the crystal structure, the authors Ibragimova, Chuvashova et al. estimated the band gap by *ab initio* calculations at different pressure points and discuss a possible use as a deep-ultraviolet birefringent material.

Two perovskite related silicates, Fe_0.5_Mg_0.5_Al_0.5_Si_0.5_O_3_ and FeMg_0.5_Si_0.5_O_3_ are reported by Koemets et al., which belong to the bridgmanite mineral class, comprising the most abundant minerals in the Earth’s lower mantle. The authors observed a spin transition for Fe^3+^ in the iron rich phase at pressures higher than 40 GPa.

Glazyrin et al. report on the synthesis of orthorhombic BiN (space group *Pbcn*) from the reaction of the two pnictogens bismuth and nitrogen at pressures above 40 GPa. Furthermore, the authors studied the phase transition and compressibility of the phases during decompression. *Ab initio* calculations were performed to support the characterization of the different BiN polymorphs.

Spektor et al. report on the impact of pressure in the ternary Na-Si-H system by computational structure prediction and *in situ* synchrotron diffraction studies. Various hypervalent hydridosilicate phases Na_*m*_SiH_(4+*m*)_ (*m* = 1–3) at comparatively low pressures of 0–20 GPa are expected, which could potentially be interesting in terms of superconductivity, ion conductivity, and hydrogen storage.

Investigations on the Dy-C system by Akbar et al. reveal the novel compounds Dy_4_C_3_ and Dy_3_C_2_, obtained in a laser-heated diamond anvil cell by means of *in situ* single-crystal synchrotron X-ray diffraction. The corresponding sesquicarbide, Dy_2_C_3_, previously only known at ambient conditions, can also be obtained at such drastic conditions.

All contributions of this Research Topic clearly demonstrate, how “extreme syntheses” can facilitate the formation of hitherto unknown compounds, enrich the structural chemistry in the whole range from seemingly simple binary phases to complex multinary materials, and expand our general knowledge and understanding of chemistry.

